# Association between BMI trajectories from childhood to early adulthood and the carotid intima-media thickness in early adulthood: Tehran lipid and glucose study

**DOI:** 10.1186/s12889-023-17184-4

**Published:** 2023-11-13

**Authors:** Amirhosein Seyedhoseinpour, Maryam Barzin, Maryam Mahdavi, Majid Valizadeh, Fereidoun Azizi, Farhad Hosseinpanah

**Affiliations:** 1 Obesity Research Center, Research Institute for Endocrine Sciences, Shahid Beheshti University of Medical Sciences, Tehran, Iran; 2 Endocrine Research Center, Research Institute for Endocrine Sciences, Shahid Beheshti University of Medical Sciences, Tehran, Iran

**Keywords:** Obesity, BMI trajectory, Waist circumference, Carotid intima media thickness, Atherosclerosis

## Abstract

**Background and aims:**

Childhood and adolescence overweight/obesity is an important predictor of obesity and increased long-term cardiometabolic abnormalities in adulthood. In this study, we aimed to investigate the association of body mass index (BMI) and waist circumference (WC) trajectories among children and adolescents with adulthood carotid intima-media thickness (cIMT) as a determinant of subclinical atherosclerosis.

**Methods:**

In this prospective cohort study, 1265 participants aged 3 to 18 were followed up for 18 years. By using Latent Class Growth Analysis, three groups of BMI and WC trajectory were defined; low stable, moderate-increasing, and high-increasing. Linear and logistic regression analysis were used to investigate the association of each lifetime BMI and WC trajectory group with cIMT.

**Results:**

Although the high-increasing BMI trajectory group was significantly associated with higher cIMT (ß=0.0464, P < 0.001), moderate-increase was not (ß=0.0096, P = 0.102); in reference to the low-stable BMI trajectory group. Among WC trajectory groups, both moderate- (ß=0.0177, P = 0.006) and high-increasing (ß=0.0533, P < 0.001), in reference to the low-stable group, were significantly associated with higher cIMT. The results did not change after adjustment for baseline BMI. The ORs of high-increasing BMI, moderate-increasing WC, and high-increasing WC trajectories were 3.24, 1.92, and 3.29, respectively for high cIMT.

**Conclusion:**

Our study resulted that a high-increasing trajectory of childhood BMI and moderate- and high-increasing trajectories of childhood WC are associated with higher cIMT and higher risk of high-cIMT. Regular monitoring and screening of BMI and WC trajectory from childhood may improve identifying individuals with high risks of cardiovascular disease, more accurately.

## Introduction

Obesity is known as a major health challenge worldwide [1]. The prevalence of overweight and obesity has increased substantially among both adults and adolescents in recent decades, which is reported to affect up to 30% of adolescents in some countries [2, 3]. In a national sample of Iranian students, aged 6–18 years, the prevalence of obesity was 13.6% and 10.1% among men and women, respectively [4]. Moreover, the prevalence of overweight and obesity among Tehranian subjects aged 3–18 years in the Tehran Lipid and Glucose Study (TLGS) was 15.4% and 6.6% respectively [5]. Childhood and adolescence obesity is an important predictor of obesity in adulthood [6, 7]. Men and women with a positive history of obesity in youth, are five and nine times more likely to have adulthood obesity, compared to healthy-weight youth, respectively [8]. Additionally, childhood and adulthood overweight/obesity are linked to not only adulthood obesity, but also to increased long-term cardiometabolic abnormalities and adverse cardiovascular disease (CVD) risk [9–14].

Body mass index (BMI) is dynamic and changes over time. Studies that measure childhood BMI in a single point, ignore the dynamic changes of BMI over time, which can be taken into account through utilizing BMI trajectories [15, 16]. Moreover, along with the intensity of obesity, the duration of obesity is an independent risk factor for cardiometabolic health [17, 18].

Studies showed that high BMI trajectories during childhood are associated with high blood pressure [19–21], type 2 diabetes mellitus (DM) [20, 22], dyslipidemia [21, 23, 24], altered intima-media thickness (IMT) and left ventricle mass index (LVMI) [25]. Also higher waist circumference (WC) trajectories are linked to higher incidence of CVD [26], DM [27]and hypertension [28].

Carotid intima-media thickness (cIMT) is a sensitive and noninvasive modality in subclinical atherosclerosis detection and quantification, [29] which is also considered as a predictive factor of cardiovascular incident [30, 31].

There are few studies [25, 32], that evaluated the association of BMI or WC trajectories and cIMT, and also among those, the effect of BMI and WC trajectories during childhood on cIMT alteration during adulthood is not well defined. In this study, we aimed to investigate the association of BMI and WC trajectories among children and adolescents with adulthood cIMT as a determinant of subclinical atherosclerosis for the first time in the Tehranian population.

The objectives of this study can give a better understanding of childhood obesity which can lead to improving screening and intervention methods during childhood and adolescence to reduce the burden of cardiovascular diseases.

## Methods and material

### Study population

In this prospective cohort study, all subjects were driven from the Tehran Lipid and Glucose Study (TLGS), a large-scale population-based prospective cohort study to determine risk factors and outcomes of non-communicable diseases [33]. In the TLGS, a total of 15,005 participants aged ≥ 3 years were recruited in the baseline cross-sectional survey (1999–2001) using a multistage random sampling method in district 13 of Tehran, capital of Iran, which to date has been followed up at three years intervals from 1999 to 2019 to update the demographic and lifestyle data, clinical and biochemical status, and anthropometric examinations. Prospective follow-up surveys were carried out from 2002 to 2005 (phase II), 2006 to 2008 (phase III), 2009 to 2011 (phase IV), 2012 to 2015 (phase V) and 2016 to 2019 (phase VI). Of all 2641 subjects with 3 to 18 years of age in phase I and II of the TLGS with available date in phase V or VI, 1455 participants could be recruited to go through cIMT measurements, which was conducted between February 2017 and October 2019. After the exclusion of subjects with cancer, pregnancy, metabolic syndrome, chronic use of corticosteroids, and extreme values of BMI (exceeding ± 3SD), 1265 were enrolled.

The study protocol conforms to the ethical guidelines of the 1975 Declaration of Helsinki as reflected in a priori approval by the Shahid Beheshti University of Medical Sciences human research committee. This study was approved by the Research Ethics Committees of the Research Institute for Endocrine Sciences (No. IR.SBMU.ENDOCRINE.REC.1399.136), and each participant provided informed consent at the beginning of the study.

### Anthropometric and laboratory assessment

The protocol and laboratory procedures of the TLGS were discussed in detail elsewhere [33]. Briefly, all data including demographics and anthropometric data were collected by trained healthcare professionals, utilizing standard questionnaires and established protocols. Weight was measured using a digital electronic scale (Seca 707; range 0.1–150 kg, Hanover, MD, USA) and was rounded to the nearest 100 g. Height was assessed using a tape stadiometer whilst subjects were barefoot in a standing position against a wall with shoulders in normal alignment and the measurements were rounded to the nearest 0.1 cm. Waist circumference (WC) was measured while the subjects were in a standing position and at the end of expiration at the narrowest level between the iliac crest and lowest rib, without any pressure on the body’s surface. BMI was calculated as $$$$Weight(kg)/{[Height(m)]^2}$$.

All blood samples were obtained at the TLGS Research Laboratory after 12 to 14 h of fasting. Then it was examined to assess fasting plasma glucose (FPG), triglyceride, total cholesterol, and high-density lipoprotein cholesterol (HDL-C). If TG < 400 mg/dl, the low-density lipoprotein cholesterol (LDL-C) was calculated using the Friedewald formula [34].

Systolic blood pressure (SBP) and diastolic blood pressure (DBP) were obtained at least two times by trained personnel utilizing a standard mercury sphygmomanometer (calibrated by the Iranian Institute of Standards and Industrial Researches) from the right brachial artery at the heart level in a sitting position and after 15 min of rest.

### Assessment of the carotid intima-media thickness

The intima-media thickness of extracranial carotid arteries was measured by two experienced radiologists using high-resolution B-mode ultrasonography equipped with a linear 7.5– 10 MHz transducer (Samsung Medison SonoAceR3 ultrasound machine). Subjects were positioned supine with their neck extended and slightly rotated to the opposite side of the examiner. The transverse plane scan was performed to assess the artery’s anatomy, locate atherosclerotic plaques (if there were any), and determine the site of maximal wall thickening in the near or far wall. Also, longitudinal scans were obtained from different angles. The measurements were obtained in a plaque-free arterial segment on an optimal grey scale of the carotid artery with a clear visualization of the far wall arterial interface while luminal content is completely anechoic. The arterial lumen was placed in the center of the image while the focal zone was set to the level of the arterial lumen by changing the depth of the scan. The hypoechoic band between the echogenic surfaces of the intima and adventitia of the arterial wall was considered cIMT. The distance of the first and second edges of the echogenic lines of the far wall was measured to assess cIMT. The left common carotid artery (LCCA) was gone through investigation in three locations and the average was considered as the final measurement. Also, sporadic measurements of the distal segment of both carotid arteries along with the carotid bulb and internal carotid artery were performed. To test the interobserver agreement, both radiologists measured cIMT in a subsample of 30 participants, consisting of 75% women with a mean age of 38.5 ± 9.2years and BMI of 25.2 ± 3.8 kg/m2. The interclass correlation coefficient (ICC) and 5% confidence interval based on a 2-way mixed-effects model were 0.79 and 0.55–0.90 respectively (ICC values between 0.75 and 0.9 indicate good reliability) [35]. In each age and gender category, the 90th percentile of cIMT is defined as high cIMT.

### Statistical analysis

Latent class growth analysis (LCGA) is a semi-parametric technique used to identify distinct subgroups of individuals following a similar pattern of change over time on a given variable. In the current analysis, this approach identifies patterns of BMI and WC, which determine individuals with similar behavioral trajectories. We applied the TRAJ procedure for Stata to build group-based multi-trajectory models via a particular application of finite mixture modeling. Model selection was made in two steps. The number of trajectory groups was determined based on the Bayesian information criterion (BIC) and significance. In the second step, we tested the various shapes of each latent class to identify the pattern of change over time (linear, quadratic, or cubic). Following the identification of the trajectory groups, each group was assigned a label regarding their pattern of BMI and WC during the follow-up phases. We examined the associations of each lifetime BMI and WC trajectory group with cIMT by linear regression models. Also, the associations of each lifetime BMI and WC trajectory group with high cIMT were examined by logistic regression models. BMI and WC trajectory group as a predictor was included in models, and the normal group was considered as a reference category. Statistical analyses were performed using Stata software, version 14.0 (Stata Corp LLC, TX, USA), and the differences with P-values less than 0·05 were considered significant (the two-tailed test).

## Results

The Study population included 1265 participants, of which 659 men (51.3%) and 616 women (48.7%) with a mean age of 10.9 ± 4.1 years. The study subjects were grouped into three BMI trajectory categories and respectively three WC trajectory categories; low-stable, moderate-increasing, and high-increasing. The BMI trajectory groups consisted of 573 (45.3%) in low-stable, 577 (45.6%) in moderate-increasing, and 115 (9.1%) in high-increasing groups. Accordingly, 463 (36.8%), 598 (47.6%), and 196 (15.6%) belonged to low-stable, moderate-increasing, and high-increasing groups of WC trajectory, respectively. In Table 1, the baseline characteristics of the subjects were compared between three BMI trajectory groups. There are significant differences in Anthropometric variables, SBP, DBP, FPG, and lipid profile between the three categories. Moreover, the cIMT showed a significant increase from low-stable to moderate-increasing and then to high-increasing of the BMI trajectories (0.54 ± 0.093 mm in low-stable, 0.56 ± 0.099 in moderate-increasing and 0.59 ± 0.11 in high-increasing group). Correspondingly, as shown in Table 2, the differences of all variables between WC trajectory groups were also significant, along with the significant difference in cIMT with an increase from low-stable to moderate-increasing and then to high-increasing of WC trajectory categories (0.54 ± 0.089 mm in low-stable, 0.55 ± 0.096 in moderate-increasing and 0.58 ± 0.11 in high-increasing group).

**Table 1:** Baseline characteristics of BMI trajectory groups

	Low-stable (n = 573)	Moderate-increasing (n = 577)	High-increasing (n = 115)	Total (n = 1265)	*P* -Value
Men, N (%)	277 (48.3)	307 (53.2)	65 (56.5)	649 (51.3)	0.129
Age, year)	9.5 ± 4.1	11.9 ± 3.7	12.7 ± 3.5	10.9 ± 4.1	< 0.001
BMI, kg/m^2^)	16.2 ± 2.6	19.7 ± 3.3	24.9 ± 4.3	18.6 ± 4.1	< 0.001
Waist circumference (cm)	58.5 ± 8.1	66.9 ± 9.7	78.8 ± 12.2	64.5 ± 11.1	< 0.001
SBP,mmHg)	101.5 ± 11.2	104.7 ± 11.2	111.6 ± 13.3	103.9 ± 11.8	< 0.001
DBP (mmHg)	69.1 ± 10.1	70.6 ± 8.8	73.9 ± 10.2	70.2 ± 9.6	< 0.001
FPG (mg/dl)	85.9 ± 8.7	88.4 ± 7.8	88.3 ± 8.2	87.3 ± 8.3	< 0.001
Total Cholesterol (mg/dl)	167.2 ± 29.6	170.2 ± 31.5	181.0 ± 37.8	169.9 ± 31.5	< 0.001
HDL-C (mg/dl)	46.0 ± 10.9	43.6 ± 10.5	39.8 ± 9.1	44.3 ± 10.7	< 0.001
Triglycerides (mg/dl) ^†^	84.0 (65.5–110.0)	97.0 (72.0-130.0)	117.0 (92.0-188.0)	86.0 (66.0-110.0)	< 0.001
cIMT (mm)	0.544 ± 0.093	0.555 ± 0.099	0.592 ± 0.113	0.553 ± 0.096	< 0.001

BMI, body mass index; cIMT, carotid intima-media thickness; DBP, diastolic blood pressure; FPG, fasting plasma glucose; HDL-C, high-density lipoprotein cholesterol; HTN, hypertension; LDL-C, low-density lipoprotein cholesterol; SBP, systolic blood pressure; TG, triglyceride

Data are given as the mean (SD) or median (IQ 25–75) unless otherwise indicated (^†^)

**Table 2:** Baseline characteristics of WC trajectory groups

	Low-stable (n = 464)	Moderate-increasing (n = 598)	High-increasing (n = 196)	Total (n = 1258)	*P -*Value
Men, N (%)	166 (35.8)	328 (54.8)	152 (77.6)	646 (51.4)	0.104
Age, year)	8.9 ± 3.9	11.8 ± 3.7	13.1 ± 3.2	10.9 ± 4.0	< 0.001
BMI, kg/m^2^)	16.2 ± 2.8	18.9 ± 3.3	23.4 ± 4.2	18.6 ± 4.1	< 0.001
Waist circumference (cm)	57.1 ± 7.4	65.4 ± 8.9	77.1 ± 11.3	64.5 ± 11.1	< 0.001
SBP,mmHg)	101.5 ± 10.9	103.9 ± 11.2	110.0 ± 12.9	104.0 ± 11.7	< 0.001
DBP (mmHg)	68.8 ± 10.2	70.5 ± 9.0	72.8 ± 9.6	70.3 ± 9.6	< 0.001
FPG (mg/dl)	85.6 ± 8.8	87.9 ± 8.0	89.2 ± 7.6	87.3 ± 8.3	< 0.001
Total Cholesterol (mg/dl)	169.8 ± 28.9	167.9 ± 31.4	175.8 ± 37.0	169.8 ± 31.6	0.012
HDL-C (mg/dl)	46.2 ± 11.1	44.0 ± 10.6	49.6 ± 8.8	44.3 ± 10.7	< 0.001
Triglycerides (mg/dl) ^†^	84.0 (65.5-113.5)	92.0 (71.0-120.0)	115.0 (83.0-166.0)	87.0 (66.0-104.0)	< 0.001
cIMT (mm)	0.543 ± 0.089	0.553 ± 0.096	0.579 ± 0.105	0.553 ± 0.095	< 0.001

BMI, body mass index; cIMT, carotid intima-media thickness; DBP, diastolic blood pressure; FPG, fasting plasma glucose; HDL-C, high-density lipoprotein cholesterol; HTN, hypertension; LDL-C, low-density lipoprotein cholesterol; SBP, systolic blood pressure; TG, triglyceride

Data are given as the mean (SD) or median (IQ 25–75) unless otherwise indicated (^†^)

The linear regression analysis for the association of BMI and WC trajectories with cIMT is depicted in Table 3. Among BMI trajectories, the association between cIMT and the high-increasing group in contrast to the reference group is significant in the unadjusted model and also after adjustment for age, sex, SBP, DBP (model 1), and additionally baseline BMI (Model 2). In contrast, the moderate-increasing group of BMI trajectory is not significantly associated with cIMT in none of the models (p = 0.063 in the unadjusted model; p = 0102 in model 1; p = 0.208 in model 2). Also, among WC trajectories, the high-increasing group is significantly associated with cIMT in all models. Accordingly, although the moderate-increasing group of WC trajectory does not have a significant association with cIMT in the unadjusted model (p = 0.122), it is significantly associated after adjustment for the abovementioned variables in models 1 and 2 (p = 0.006 and 0.040, respectively).

**Table 3:** Linear regression coefficients for the association of BMI and WC trajectories with cIMT

	BMI Trajectories			WC Trajectories		
	ß	SE	*P*-Value	ß	SE	*P* -Value
**Unadjusted Model**						
Moderate-increasing	0.0104	0.0056	0.063	0.0091	0.0059	0.122
High-increasing	0.0480	0.0097	< 0.001	0.0357	0.0081	< 0.001
**Model 1**						
Moderate-increasing	0.0096	0.0059	0.102	0.0177	0.0064	0.006
High-increasing	0.0464	0.0101	< 0.001	0.0533	0.0091	< 0.001
**Model 2**						
Moderate-increasing	0.0081	0.0064	0.208	0.0145	0.0070	0.040
High-increasing	0.0422	0.0126	0.001	0.0433	0.0116	< 0.001

SE, standard error

Model 1 = age, sex, systolic blood pressure, diastolic blood pressure

Model 2 = Model 1 + Baseline BMI (for BMI Trajectories) / Baseline WC (for WC Trajectories)

The logistic regression analysis along with the odds ratio (OR) for the association of BMI and WC trajectories with high cIMT is depicted in Table 4. The ORs of the high-increasing group of BMI trajectories for high cIMT were 3.6, 3.2, and 3.1 in the unadjusted model, model 1 (adjusted for age, sex, systolic blood pressure, diastolic blood pressure) and model 2 (model 1 + baseline BMI), respectively. Among WC trajectories, the ORs of the moderate-increasing group in association with high cIMT ranged from 1.78 to 1.95, and in the high-increasing group of WC trajectories, it ranged from 3.3 to 3.9.

**Table 4:** Odds ratios for the association of BMI and WC trajectories with high cIMT*

	BMI Trajectories			WC Trajectories		
	OR	95% CI	*P* -Value	OR	95% CI	*P* -Value
**Unadjusted Model**						
Moderate-increasing	1.5058	(0.9942, 2.2808)	0.053	1.9486	(1.2118, 3.1335)	0.006
High-increasing	3.6043	(2.0895, 6.2174)	< 0.001	3.7904	(2.2178, 6.4780)	< 0.001
**Model 1**						
Moderate-increasing	1.3886	(0.8997, 2.1434)	0.138	1.9289	(1.1543, 3.2234)	0.012
High-increasing	3.2398	(1.7972, 5.8404)	< 0.001	3.9015	(2.0894, 7.2852)	< 0.001
**Model 2**						
Moderate-increasing	1.3851	(0.8672, 2.2121)	0.173	1.7835	(1.0287, 3.0920)	0.039
High-increasing	3.1353	(1.4589, 6.7379)	0.003	3.2905	(1.5167, 7.1390)	0.003

* High cIMT is defines as 90th percentile of cIMT in each age and gender categories

OR, odds ratio

Model 1 = age, sex, systolic blood pressure, diastolic blood pressure

Model 2 = Model 1 + Baseline BMI (for BMI Trajectories) / Baseline WC (for WC Trajectories)

## Discussion

In the current study conducted among Iranian adolescents, we investigated the association of developmental patterns of BMI and WC with the adulthood cIMT as a contributor to subclinical atherosclerosis. The results of our study demonstrated that subjects of the high-increasing BMI trajectory group have significantly higher cIMT in adulthood. Accordingly, among WC trajectory groups, subjects of moderate-increasing and high-increasing groups have both higher cIMT in contrast to the low-stable WC trajectory group. Compared to the low-stable BMI trajectory, for high cIMT, the high-increasing trajectory increased the risk by approximately 3-fold. Also, the moderate-increasing and high-increasing trajectories of WC increased the risk of high adulthood cIMT, approximately 2- and 3-fold, respectively, in contrast to the low-stable WC trajectory group.

The subjects of our study were grouped into three categories based on BMI trajectory and WC trajectory, which represented the substantial heterogenicity of BMI and WC dynamic changes during over 20 years of follow-up through childhood to early adulthood. Several other studies with the same approach, utilizing latent class modeling, reached different numbers of WC and BMI trajectory groups, which were labeled to the pattern of dynamic changes and ranged from two to 6 groups, in different studies [25, 26, 36–40]. Also, the same number of BMI trajectory groups in our study was defined in some previous studies with the follow-up of subjects from childhood to adulthood [25, 37, 38, 40].

Childhood overweight/ obesity is defined to be a major risk factor for adulthood obesity. The results of a systematic review study by Singh et al. [6] showed that overweight and obese youth have an increased risk of being overweight during adulthood. Preventing overweight and obesity from an early age should be considered a major public health priority because, not only once an individual becomes obese, they are unlikely to return to normal body weight, but also childhood overweight and obesity can be an independent risk factor of cardiometabolic health during adulthood [17, 18]. Also, the cardiovascular consequences of obesity are shown to be cumulative and the duration of obesity is a strong predictor of CVD outcomes [13, 26, 41–43].

The dynamic and cumulative consequences of overweight/ obesity, as reflected in BMI or WC trajectory, are associated with many cardiometabolic risk factors and also cardiovascular outcomes. In the study of Blond et al. [38] higher BMI trajectories in childhood were associated with worse HDL, glucose homeostasis, total cholesterol, and triglyceride levels in adulthood. In another study on the Chinese population, the ratio risk of hypertension, type 2 diabetes mellitus, high-risk triglycerides, and high-risk HDL was more than 3.0 during adulthood among the high-increasing childhood BMI trajectory group [37]. Among other cardiometabolic risk factors, higher risk of hypertension incidence [20, 44, 45], metabolic syndrome [40], unfavorable levels of trunk fat [46], insulin [46], HDL [21, 46], left ventricular mass [25, 46], high plasma glucose [20] and incidence of diabetes, in adulthood are significantly associated with higher BMI trajectories during childhood.

Furthermore, among cardiovascular outcomes, the correlations between high BMI trajectories in adulthood and elevated lifetime risk of cardiovascular disease, stroke, and myocardial infarction were observed in a previous study [43]. In the study of Tirosh et al., BMI trajectories of adolescence to adulthood were measured, in which the hazard ratio of angiography-proven coronary heart disease was 5.42 in elevated adolescence BMI trajectory in multivariate model adjustment that stayed significant after adjustment for BMI at adulthood [22]. Correspondingly, WC trajectory is also correlated to the incidence of hypertension [27], level of diastolic and systolic blood pressure [47], the incidence of diabetes [28], and the incidence of cardiovascular disease [26].

The adverse effect of youth overweight/ obesity is also reflected in adult pre-atherosclerosis and it may not be reversible even with weight normalization later in life [48]. The results of our study indicated that both WC and BMI trajectories from childhood to early adulthood are predictors of cIMT in adulthood. The study of Buscot et al. [39] demonstrated that trajectories of worsening or persisting obesity from childhood to adulthood are associated with higher cIMT levels in adulthood. Even participants who resolved their childhood overweight/ obesity had an elevated risk of increased cIMT in adulthood [39]. Consistent with this study, our results specify that higher BMI and WC trajectories have a higher risk of high cIMT in adulthood (OR = 1.4, p = 0.17 and OR = 3.1, p = 0.003 for moderate-increasing and high-increasing BMI trajectories, respectively; and OR = 1.8, p = 0.039, and OR = 3.3, p = 0.003 for moderate-increasing and high-increasing WC trajectories, respectively, in contrast to low-stable trajectory). Although the moderate- and high-increasing WC trajectory group had significantly higher cIMT, this association is only significant among the high-increasing BMI trajectory group, but not moderate-increasing BMI trajectory. In the study of Hao et al. [25], with the same BMI trajectory groups as our study, childhood BMI trajectory was significantly associated with adulthood cIMT after adjustment in a multivariate model for covariates (ß=0.014, p = 0.043 and ß=0.034, p = 0.001 for moderate increasing and high-increasing BMI trajectory, respectively); but it does not remain significant after adjustment for baseline BMI (p = 0.354 for moderate increasing; p = 0.250 for high-increasing BMI trajectory). In our study, the significance of the association between BMI/ WC trajectories and cIMT does not alter after adjustment for baseline BMI (ß=0.014, p = 0.043 and ß=0.034, p = 0.001 for moderate increasing and high-increasing BMI trajectory, respectively).

The current study has several strengths; the study is longitudinal and a follow-up long enough to examine the heterogeneity in BMI trajectories from childhood until early to mid-adulthood that allowing us to evaluate the effect of trajectories on endpoint cIMT. Second, LCGA, as a standard method to identify trajectories, involves several criteria in the identification of qualitatively distinct trajectories, selecting the best fitting model and predicting individual class membership from the available data and can deal with missing at random assumption. Regarding limitations of the study, our data were obtained from a metropolitan city of Iran and may not be national or to other population representatives. Second, only participants with available cIMT data were eligible for the study. Third, due to the limited sample size, we might not be able to capture all trajectory patterns. More studies with large sample sizes should be performed to confirm our results.

In conclusion, our study showed that a high-increasing trajectory of childhood BMI and moderate- high-increasing trajectories of childhood WC are associated with higher cIMT and higher risk of high cIMT. These data suggested that regular monitoring and screening of BMI and WC trajectory from childhood may improve the identification of individuals with high risks of cardiovascular disease and implement more accurate targeted strategies of prevention.

## Data Availability

The datasets used and analyzed during the current study are available from the corresponding author on reasonable request.
